# Deep learning-driven ultrasound-assisted diagnosis: optimizing GallScopeNet for precise identification of biliary atresia

**DOI:** 10.3389/fmed.2024.1445069

**Published:** 2024-10-08

**Authors:** Yupeng Niu, Jingze Li, Xiyuan Xu, Pu Luo, Pingchuan Liu, Jian Wang, Jiong Mu

**Affiliations:** ^1^College of Information Engineering, Sichuan Agricultural University, Ya’an, China; ^2^Artificial Intelligence Laboratory, Sichuan Agricultural University, Ya’an, China; ^3^College of Veterinary Medicine, Sichuan Agricultural University, Chengdu, China; ^4^People’s Hospital of Ya’an City, Sichuan Province, Ya’an, China; ^5^Department of Neurology, The Affiliated Hospital, Southwest Medical University, Luzhou, Sichuan, China

**Keywords:** biliary atresia (BA), deep learning, ultrasound diagnosis, feature extraction, clinical application

## Abstract

**Background:**

Biliary atresia (BA) is a severe congenital biliary developmental abnormality threatening neonatal health. Traditional diagnostic methods rely heavily on experienced radiologists, making the process time-consuming and prone to variability. The application of deep learning for the automated diagnosis of BA remains underexplored.

**Methods:**

This study introduces GallScopeNet, a deep learning model designed to improve diagnostic efficiency and accuracy through innovative architecture and advanced feature extraction techniques. The model utilizes data from a carefully constructed dataset of gallbladder ultrasound images. A dataset comprising thousands of ultrasound images was employed, with the majority used for training and validation and a subset reserved for external testing. The model’s performance was evaluated using five-fold cross-validation and external assessment, employing metrics such as accuracy and the area under the receiver operating characteristic curve (AUC), compared against clinical diagnostic standards.

**Results:**

GallScopeNet demonstrated exceptional performance in distinguishing BA from non-BA cases. In the external test dataset, GallScopeNet achieved an accuracy of 81.21% and an AUC of 0.85, indicating strong diagnostic capabilities. The results highlighted the model’s ability to maintain high classification performance, reducing misdiagnosis and missed diagnosis.

**Conclusion:**

GallScopeNet effectively differentiates between BA and non-BA images, demonstrating significant potential and reliability for early diagnosis. The system’s high efficiency and accuracy suggest it could serve as a valuable diagnostic tool in clinical settings, providing substantial technical support for improving diagnostic workflows.

## 1 Introduction

Biliary atresia (BA) is a severe congenital anomaly of the biliary system that poses a significant threat to neonatal health. It is characterized by the obstruction of parts or all of the biliary tract, which prevents bile from flowing into the duodenum. This obstruction results in bile accumulation in the liver, leading to progressive liver damage, biliary cirrhosis, and ultimately liver failure. Without timely intervention, liver transplantation becomes necessary, posing a life-threatening risk to affected infants (1). The exact etiology of BA remains unclear, but it is believed to involve a combination of genetic predisposition and environmental factors, potentially including viral infections and immune-mediated damage during fetal development (1, 2). Early and accurate diagnosis is crucial for initiating treatment plans, preventing complications, reducing mortality, and improving patient prognosis. Interventions such as the Kasai portoenterostomy, which involves surgically bypassing the blocked bile ducts, can be life-saving if performed early, but the success of such procedures diminishes with delays in diagnosis and treatment (1). Additionally, advancements in imaging techniques, such as the development of novel NIR-II fluorescent probes, have shown promise in improving the visualization of biliary structures and aiding in the early detection of BA (2).

Ultrasound imaging, particularly abdominal ultrasound, is widely employed as an initial screening tool for BA in pediatrics due to its non-invasive, convenient, cost-effective, and real-time characteristics (3). However, the ultrasound features of BA are often atypical, manifesting only as gallbladder enlargement, bile duct dilation, or enhanced liver echogenicity (4). This demands high image recognition skills from diagnosticians and vigilance towards subtle pathological changes. The heavy reliance on operator experience and subjective interpretation of pathological features compromises diagnostic consistency and accuracy (5). Traditional diagnostic strategies, including clinical symptom monitoring (such as the duration and appearance of jaundice) (6), biochemical markers (such as direct bilirubin levels) (7, 8), and imaging examinations, although informative, frequently fail to achieve optimal sensitivity and specificity in the early stages of the disease, delaying timely treatment (7). Manual interpretation of ultrasound images is not only time-consuming but also prone to variability due to operator experience and fatigue, increasing diagnostic inconsistency (9, 10).

Recent advancements in elastography, including transient elastography and shear wave elastography, have been explored to improve the diagnostic accuracy for liver fibrosis and BA (4, 5, 9). Despite these advancements, significant challenges remain in differentiating BA from other causes of neonatal jaundice using conventional ultrasound techniques (9). Studies have shown that combining gray-scale ultrasound with elastography may enhance diagnostic performance, but this approach still heavily depends on the operator’s expertise and experience (9).

Furthermore, current diagnostic methods for BA, such as the infant stool color card screening program and newborn screening for direct or conjugated bilirubin measurements, have demonstrated varying degrees of success in early detection. These methods, while useful, often lack the necessary sensitivity and specificity to reliably identify BA in its early stages, leading to delays in diagnosis and treatment (6, 7).

In recent years, artificial intelligence (AI) technologies, particularly deep learning (DL), have introduced groundbreaking advancements in medical imaging analysis for BA diagnosis (10). Convolutional neural networks (CNNs), a fundamental component of DL, have demonstrated exceptional image analysis capabilities. CNNs automatically extract complex visual patterns and structural information from raw images through multi-level feature learning, thereby identifying disease characteristics (11). Studies indicate that CNNs, when diagnosing diseases such as tumors and cardiovascular conditions, achieve accuracy levels surpassing those of human experts, providing more objective and accurate support for clinical decision-making (12–14). Given the urgency of early BA diagnosis and the challenges in interpreting ultrasound images, developing objective, efficient, and accurate AI-based diagnostic tools has become a research focus. These tools aim to leverage CNNs and other technologies to automatically identify subtle pathological changes in ultrasound images, reduce dependence on operator experience, improve diagnostic accuracy and consistency, and thereby promote early identification and intervention for BA, ultimately enhancing patient outcomes (15).

**Figure 1:**
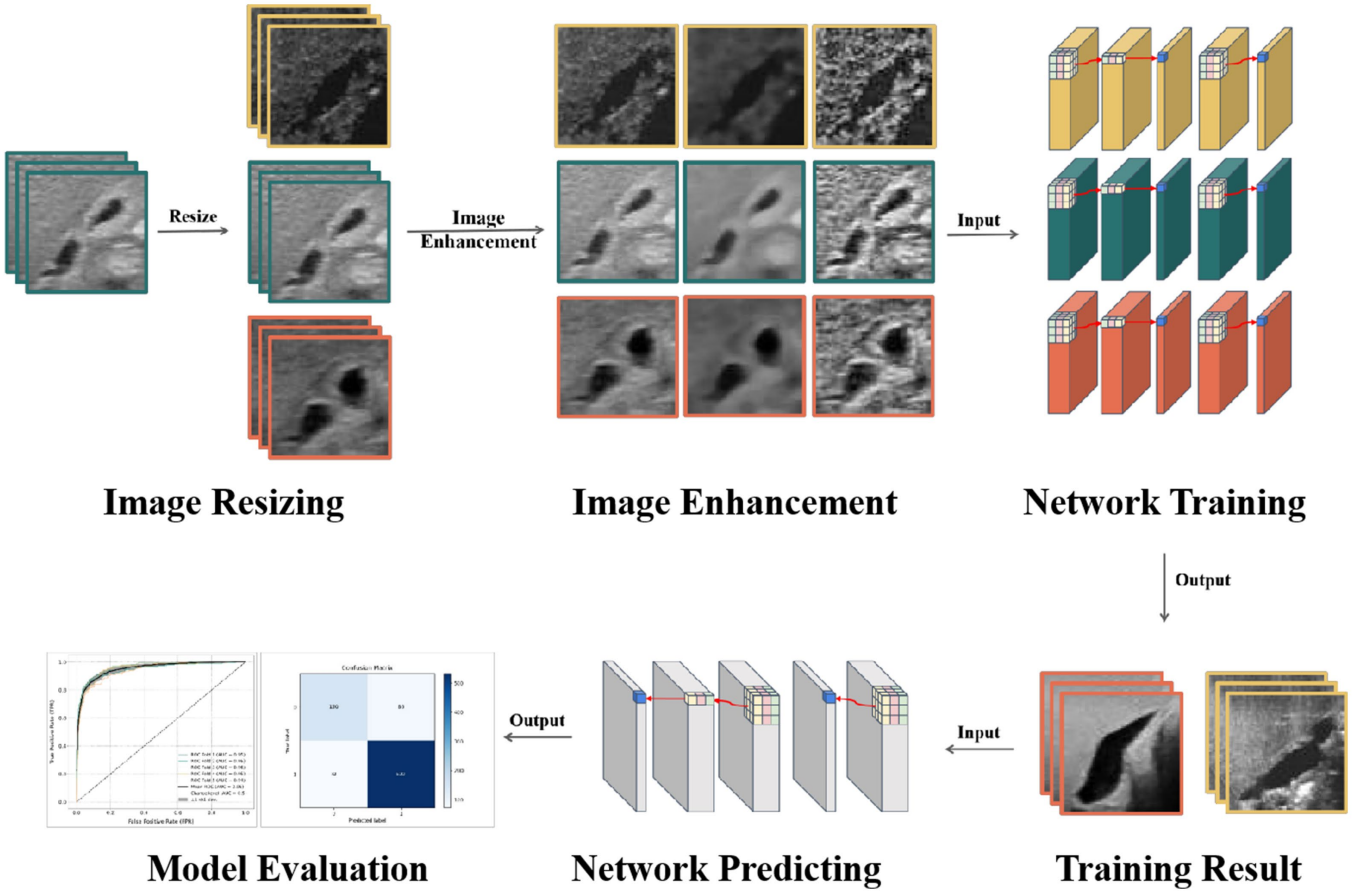
Workflow.

This study aims to develop a deep learning model specifically designed for the automatic diagnosis of BA in ultrasound gallbladder images. By focusing on the optimization of feature extraction and image enhancement techniques, this study seeks to overcome the limitations of existing models, thereby enhancing diagnostic accuracy and generalization capabilities (Figure 1). Specifically, the innovations of this study include:

1. Model design innovation: Developing an integrated CNN model specifically targeting the identification of changes in gallbladder and bile duct structures, enhancing the model’s sensitivity and specificity. The design focuses on optimizing structural and feature extraction levels to better identify and interpret BA characteristics.
2. Image enhancement and optimization: Utilizing advanced image enhancement techniques to improve the model’s ability to recognize subtle pathological changes, ensuring consistent performance across images of varying quality.
3. Rigorous model validation: Implementing stringent model validation strategies, including five-fold cross-validation and independent external test set validation, to ensure the model’s stability and generalizability across different datasets, further verifying the model’s reliability and clinical application potential.

## 2 Related work

Biliary atresia (BA) is a severe congenital liver condition that leads to bile duct obstruction in neonates, necessitating early and accurate diagnosis to prevent liver failure. Traditional diagnostic methods such as liver biopsy and intraoperative cholangiography (IOC) are considered gold standards due to their high sensitivity and specificity. Liver biopsy, despite its high diagnostic performance, is invasive and prone to sampling errors, and requires expert pathological interpretation (16). IOC, another gold standard, can only be performed by pediatric surgeons and involves significant risks (17). Non-invasive methods like hepatobiliary scintigraphy and MRI offer alternatives but have limitations, including low specificity and the need for sedation (12, 17).

Ultrasound (US) is widely used as a non-invasive diagnostic tool for BA due to its feasibility and real-time imaging capabilities. Key ultrasound features for BA diagnosis include the triangular cord sign and gallbladder abnormalities. The triangular cord sign, indicating fibrosis of the extrahepatic bile duct, has high specificity but variable sensitivity (12, 15). Park et al. (18) emphasized the importance of the triangular cord sign in diagnosing BA and compared the effectiveness of ultrasonography, hepatobiliary scintigraphy, and liver needle biopsy, highlighting the varying diagnostic performance of these methods. Despite its advantages, the diagnostic performance of ultrasound heavily depends on the operator’s expertise, leading to variability and potential misdiagnosis, particularly in settings with less experienced radiologists (12, 19). This variability highlights the need for more consistent and accurate diagnostic tools.

Recent advancements in deep learning, particularly convolutional neural networks (CNNs), have shown significant promise in medical imaging. Gulshan et al. (20) demonstrated that CNNs could achieve dermatologist-level accuracy in skin cancer detection, showcasing the potential of AI to surpass human experts in diagnostic tasks. In the context of BA, Liu et al. (21) developed an ensemble deep learning model that outperformed human experts in diagnosing BA from sonographic images, achieving high accuracy and robustness across multiple centers. Their model utilized advanced feature extraction techniques and demonstrated a significant improvement in diagnostic performance, with a reported sensitivity of 93.1% and specificity of 93.9%. However, the study’s reliance on high-quality sonographic images and the increased computational complexity of the ensemble model present challenges for its practical application in diverse clinical settings.

Several other studies have further validated the effectiveness of deep learning in medical diagnostics. Rajpurkar et al. (22) employed a deep learning model to assist in the diagnosis of diabetic retinopathy, achieving sensitivity and specificity comparable to ophthalmologists. Similarly, Zhou et al. (23) developed a deep learning algorithm for the detection of diabetic retinopathy, which showed a high level of accuracy in a large dataset. Additionally, Gu et al. (24) used a deep learning model to interpret chest radiographs for various pathologies, demonstrating that their model could achieve radiologist-level performance.

In the context of BA, other notable studies include Caponcelli et al. (25), who developed an interpretable AI-based app to assist inexperienced radiologists in diagnosing BA from sonographic gallbladder images, achieving significant improvements in diagnostic accuracy. Another study by Zhang et al. (26) applied deep learning techniques to analyze stool color images for early detection of BA, showing high sensitivity and specificity. These studies collectively highlight the transformative potential of AI in enhancing diagnostic accuracy and consistency across various medical fields.

Despite these advancements, several challenges remain. Many existing AI models rely on high-quality imaging data and extensive preprocessing, which may not always be feasible in real-world clinical settings. Gu et al. (24) pointed out that the reliance on high-quality images limits the applicability of these models in settings with variable imaging quality. Additionally, the diversity of training datasets is often limited, affecting the generalizability of the models across different populations and imaging conditions. Caponcelli et al. (25) noted that models trained on homogeneous datasets may struggle with diverse clinical data, highlighting the need for more inclusive and representative training datasets.

In response to these challenges, this study introduces GallScopeNet, an innovative deep learning model designed to optimize feature extraction and diagnostic accuracy for BA. GallScopeNet incorporates normal convolution and coordinate attention mechanisms to enhance its ability to detect subtle pathological features in ultrasound images while maintaining computational efficiency. This approach aims to improve the robustness and generalizability of the model across diverse clinical settings. By addressing these gaps, GallScopeNet aims to provide a reliable, efficient, and non-invasive diagnostic tool for early BA detection, potentially improving clinical outcomes and reducing the burden on healthcare systems.

## 3 Materials and methods

### 3.1 Data collection

The dataset for this study was sourced from a meticulously designed specialized resource—sonographic images for biliary atresia diagnosis [Zenodo. (2021). youngyzzZ/sonographic-gallbladder-images-for-BA-diagnosis: fifth release of my project (v1.0.4). Available at: https://doi.org/10.5281/zenodo.4445734] (27). This database extensively collected and organized ultrasound image data related to BA, providing a valuable experimental foundation for this study. The dataset includes a total of 927 confirmed cases of BA and 2,778 control cases (non-BA patients). All diagnoses were confirmed through intraoperative cholangiography, percutaneous ultrasound-guided cholecystocholangiography, liver biopsy, or follow-up, ensuring the data’s reliability and accuracy. Inclusion criteria for the dataset were neonates with a clinical suspicion of biliary atresia based on initial clinical assessments and laboratory tests, with confirmation of the diagnosis through intraoperative cholangiography, percutaneous ultrasound-guided cholecystocholangiography, liver biopsy, or follow-up. Additionally, only high-quality ultrasound images that met predefined imaging standards were included. Exclusion criteria involved cases with poor-quality ultrasound images that did not meet these standards, neonates with other diagnosed liver conditions that could confound the diagnosis of biliary atresia, and patients with incomplete clinical records or lack of follow-up data to confirm the diagnosis.

### 3.2 Data preprocessing

The dataset used in this study included ultrasound images of varying quality, with differences in aspects such as contrast, noise levels, and the presence of artifacts. These variations in image quality reflect real-world clinical conditions, where factors such as differences in ultrasound equipment, patient movement during scanning, varying operator expertise, and suboptimal imaging conditions can significantly impact the final ultrasound image. Variations in contrast and the presence of noise or artifacts can obscure critical features necessary for accurate diagnosis. Additionally, artifacts such as shadowing, reverberation, and speckle noise often present in ultrasound images further complicate the analysis process.

Given these challenges, the selection process in this study was guided by the visibility of key anatomical features crucial for diagnosing biliary atresia. Images were evaluated based on their contrast quality, noise levels, and the presence of significant artifacts to ensure they did not negatively impact the diagnostic process. Specifically, images with poor contrast, excessive noise—such as speckle or electronic interference—or artifacts that could obscure or distort anatomical structures were excluded from the dataset. After applying these selection criteria, 897 confirmed cases of BA and 2,689 control cases (non-BA patients) were included in the final dataset. This selection ensured that the model was trained on images where quality issues did not significantly hinder diagnostic accuracy, thereby providing a robust foundation for developing the model without necessarily relying on only the highest-quality images.

After completing the rigorous selection process, the selected ultrasound images underwent further optimization through advanced preprocessing and enhancement techniques. All images were uniformly resized to a standard dimension of 224 × 224 pixels. This resizing preserved data integrity while promoting efficient model training and ensuring unified image information processing and feature extraction.

**Figure 2:**
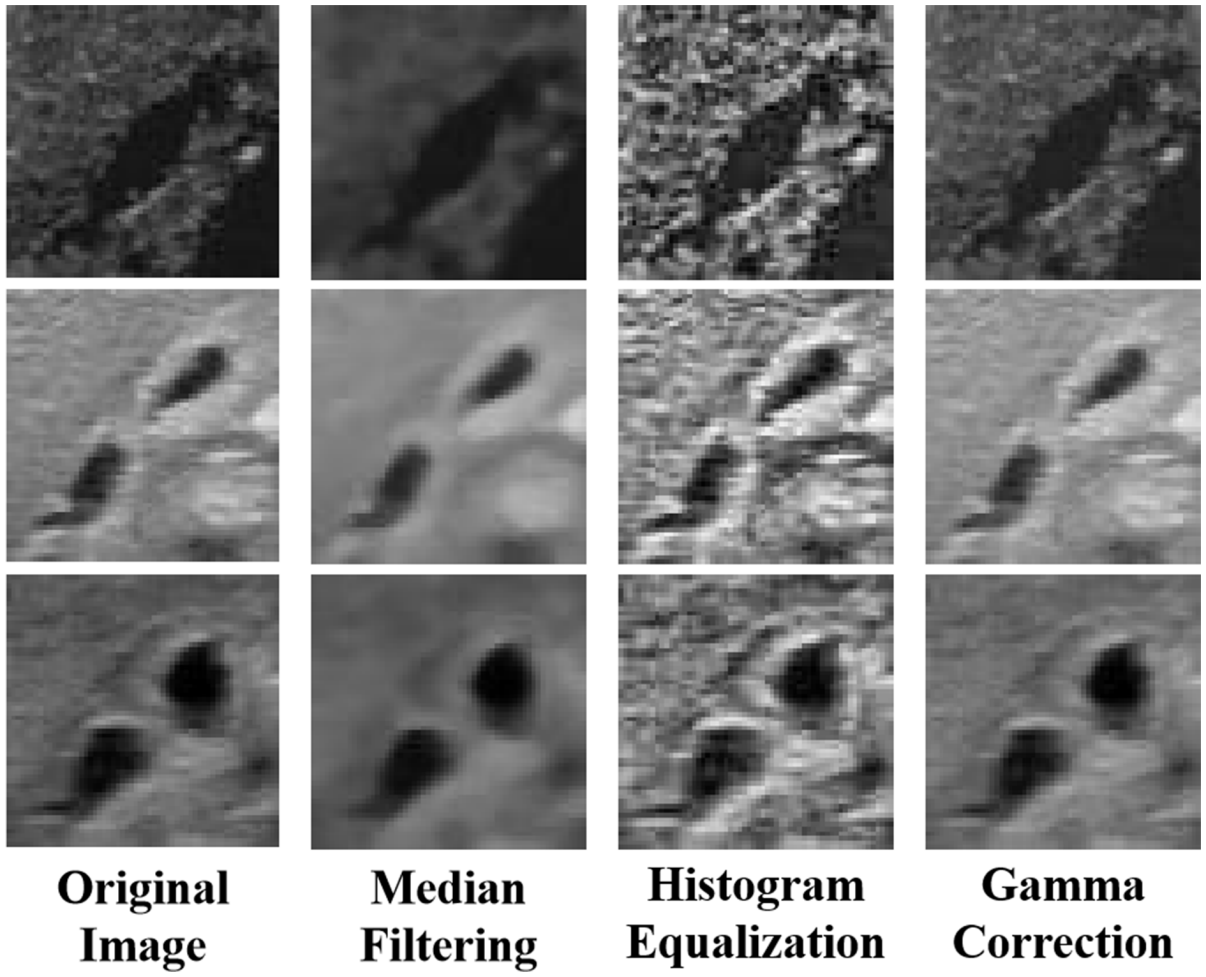
Image enhancement using adaptive histogram equalization (AHE), median filtering, and gamma correction.

Secondly, to further enhance the model’s learning performance in complex ultrasound images, a series of advanced data augmentation techniques were implemented to increase training set diversity and robustness. Adaptive histogram equalization (AHE) was used to adjust pixel brightness distribution adaptively (28), significantly enhancing contrast and making details such as gallbladder walls and internal textures more prominent, providing the model with rich information layers for learning. Median filtering effectively removed random noise from images (29), particularly ultrasound artifacts, similar to methods used for low-dose CT image denoising and enhancement in previous studies (30), preserving clear key structures like the gallbladder and bile ducts, thereby improving the model’s robustness in practical image processing and reducing misreads. Gamma correction adjusted image brightness and contrast (31), rendering overly dark or bright details visible, aiding in the recognition of subtle pathological features such as bile flow and blood flow, thereby enhancing the model’s sensitivity and specificity (Figure 2).

Additionally, a comprehensive suite of data augmentation techniques was applied to further enhance the robustness and generalizability of the model. These techniques included rotation by random angles within a specified range, ensuring the model learns to recognize anatomical structures regardless of their orientation, and scaling, where images were randomly scaled up or down to ensure the model becomes invariant to differences in image size and resolution. Flipping was applied horizontally and vertically to help the model generalize across different patient positions and imaging protocols. Random cropping of images forced the model to focus on different parts of the image, enhancing its ability to detect features that may appear in various locations. Noise injection added random noise to the images, simulating variations and imperfections that occur in real-world ultrasound imaging, thereby improving the model’s robustness to noisy or poor-quality images. Finally, elastic deformation was used to randomly deform the images, simulating the variability in tissue appearance due to different imaging conditions or patient movements, which helps the model to recognize anatomical structures under varied conditions.

These augmentation techniques were carefully chosen to address potential biases and ensure that the model encounters a wide range of variations during training. This approach not only increases the diversity of the training set but also improves the model’s ability to generalize to different clinical settings and patient populations, thus reducing the risk of overfitting to specific types of data. These meticulously designed data processing and augmentation techniques not only improved image quality but also strengthened the model’s ability to recognize pathological features of BA, laying a solid foundation for model generalization and diagnostic accuracy. Through these data preparation steps, the model effectively extracts key information from ultrasound images, providing an efficient and reliable auxiliary tool for automatic diagnosis of gallbladder and BA.

### 3.3 Model construction

Deep learning, particularly convolutional neural networks (CNNs), has revolutionized the field of medical imaging by enabling automatic and accurate analysis of visual data. CNNs are designed to learn hierarchical patterns and features from images through multiple layers of convolution.

At a high level, CNNs consist of several types of layers: convolutional layers, pooling layers, and fully connected layers. The convolutional layers apply a set of learnable filters to the input image, where each filter convolves around the input image and activates certain features such as edges, textures, and colors. This process generates feature maps that capture various aspects of the image. Pooling layers then reduce the spatial dimensions of these feature maps, retaining the most essential information while reducing the computational load and preventing overfitting. Finally, fully connected layers process these features to classify the image into predefined categories (11).

The ability of CNNs to automatically learn and extract features from raw image data makes them particularly effective for image recognition tasks. This capability is further enhanced by using multiple layers, allowing the network to understand and capture increasingly complex and abstract features at each layer (11). The hierarchical learning approach of CNNs thus enables them to excel in various medical diagnostic applications by accurately identifying and interpreting intricate patterns in medical images.

However, designing CNN models that balance efficiency and accuracy can be challenging. This study focuses on finding a balanced solution between model efficiency and accuracy to meet the needs of embedded systems for medical diagnostics (32). Based on this, a lightweight ResNet34 model incorporating the PConv (partial convolution) architecture from FasterNet was used as the baseline model (33, 34). This model is a proven efficient model designed to provide good performance even in resource-limited environments. However, to further optimize the model’s diagnostic capabilities, particularly for recognizing complex features in gallbladder images indicative of BA, a series of innovative improvement strategies were designed to enhance model performance without significantly increasing complexity.

#### 3.3.1 Improvement A: baseline (−PConv)

In optimizing the deep learning model for diagnosing BA from ultrasound gallbladder images, this study first re-examined the use of partial convolution (PConv). PConv was introduced in previous studies to reduce model complexity and computational cost (35, 36). The core idea of PConv is to apply standard convolution to only a portion of the input feature map’s channels, reducing computation and memory access frequency to improve operational efficiency. Specifically, PConv selects a small continuous portion of the feature map channels (e.g., one-quarter) for computation, while the rest remain unchanged, effectively reducing the number of floating-point operations (FLOPs) and memory access needs.

PConv modifies the traditional convolution process by focusing only on certain parts of the input, thereby reducing the computational load. This selective approach helps in speeding up the model and lowering the resource requirements, which is beneficial for applications in resource-limited environments. However, this reduction in computation might lead to a compromise in capturing detailed features necessary for high-precision tasks like diagnosing BA from ultrasound images.

Deep analysis revealed that for diagnosing diseases like BA, the subtle structural changes, morphology of the gallbladder wall, and hemodynamic features present in ultrasound images demand higher recognition capabilities from the model (37). Normal convolution, which covers all channels of the input feature map, has an inherent advantage in capturing these detailed features, despite higher computational costs and parameter counts (38). Normal convolution, through its global operation, can more fully extract inter-feature relationships and spatial contextual information, crucial for distinguishing normal and abnormal gallbladder structures.

Normal convolution involves applying a convolutional filter across the entire input feature map, ensuring that every channel and pixel is considered. This comprehensive approach allows the model to capture intricate details and spatial relationships within the image. By processing the full input, normal convolution can extract rich and complex features, making it particularly effective for identifying subtle differences between normal and abnormal gallbladder structures. This thorough feature extraction is crucial for accurate diagnosis in medical imaging, where precision is paramount.

**Figure 3:**
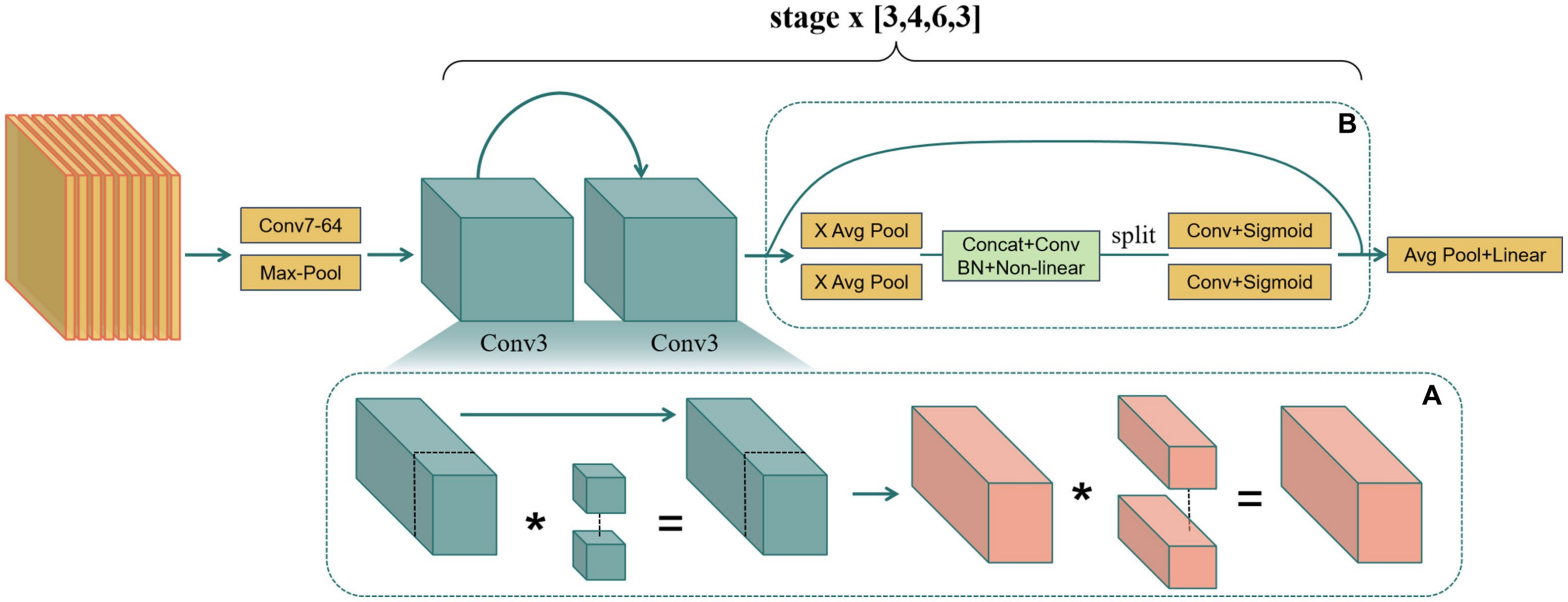
**(A)** Replacing partial convolution with normal convolution to enhance sensitivity to detailed features. **(B)** Embedding coordinate attention to optimize feature extraction.

Therefore, this study replaced the partial convolution in the baseline model with normal convolution (Figure 3A), sacrificing some model complexity to enhance performance in diagnosing BA from ultrasound images. Despite the increase in computational complexity, normal convolution’s performance advantages in ultrasound image analysis are realized, especially in capturing intricate details and dynamic structures in gallbladder images. This includes more finely analyzing the slight changes in the gallbladder wall and identifying abnormal hemodynamic features indicative of BA, critical for improving diagnostic accuracy.

#### 3.3.2 Improvement B: baseline (+CA)

Coordinate attention (CA), an innovative attention mechanism in current mobile network design, has shown significant effects in enhancing model performance (39–41). CA embeds positional information into traditional channel attention, overcoming the limitations of ignoring spatial information. Specifically, it aggregates information along the horizontal and vertical directions through two one-dimensional feature encoding processes, maintaining both long-range spatial dependencies and precise positional details. This mechanism generates direction-aware and position-sensitive attention maps that complementarily apply to the input feature map, enhancing the expression of key areas, aiding the model in accurately locating and identifying target objects.

To elaborate, traditional channel attention mechanisms focus on emphasizing important channels but often overlook the spatial relationships within the image. CA addresses this by capturing the positional information along both the horizontal and vertical dimensions. It achieves this by splitting the input feature map into horizontal and vertical directions and encoding each direction separately. This approach allows the model to maintain a global view of the spatial dependencies while preserving detailed positional information.

The CA mechanism generates attention maps that are sensitive to the direction and position of features within the image. These attention maps are then applied to the input feature map, enhancing the model’s ability to focus on and accurately identify critical areas within the image. By doing so, CA enhances the model’s feature extraction capabilities, leading to improved performance in tasks requiring detailed spatial understanding.

This study’s improvement strategy focused on integrating this efficient CA attention mechanism into the model’s basic construction units to significantly improve model performance while strictly controlling the increase in model complexity. The approach goes beyond merely adding an attention module to the existing architecture but integrates CA into the fundamental unit design, making it an inseparable part of the model architecture. The carefully designed integration scheme ensures CA operations work synergistically with basic convolution operations, jointly optimizing the feature extraction process.

In practical implementation, the existing basic unit basic block was fine-tuned, integrating CA with minimal computational cost by adding the CA mechanism after the residual connection of the basic unit. The residual connection’s fused image feature data is fed into the CA mechanism to enhance the expression of key areas (Figure 3B). This allows attention weights to guide feature learning effectively with minimal model complexity increase.

#### 3.3.3 GallScopeNet

Based on the advantages of the two improvements, this study optimized the model’s feature expression capabilities and designed the final model, GallScopeNet (Figure 3). The architecture of GallScopeNet integrates several key components and strategies to enhance its performance in diagnosing biliary atresia from ultrasound images.

Firstly, the model employs normal convolution instead of partial convolution. While this substitution results in a slight increase in the parameter count by approximately 0.1%, it significantly boosts the model’s ability to extract features from ultrasound gallbladder images. Specifically, GallScopeNet achieves an improvement of over 3% in accuracy and F1 score compared to the baseline model using partial convolution. These enhancements are crucial for identifying subtle pathological features associated with biliary atresia, demonstrating GallScopeNet’s superior performance in critical diagnostic tasks.

Secondly, GallScopeNet incorporates coordinate attention (CA) into its architecture. This innovative attention mechanism embeds positional information into the traditional channel attention framework, allowing the model to maintain high efficiency while improving feature expression. The inclusion of CA contributes to an increase of over 8% in both overall accuracy and F1 score on the test datasets. Additionally, the integration of CA adds only about 0.7% to the model’s parameter count, ensuring that GallScopeNet remains computationally efficient while significantly enhancing its diagnostic performance.

The integration of these components within GallScopeNet is designed to keep the model’s complexity and optimization latency in check. The architecture includes a series of convolutional layers with normal convolution and CA modules, arranged to maximize the extraction and utilization of critical features from the ultrasound images. The backbone of the model is based on a modified ResNet34 framework, which ensures a balance between depth and computational efficiency.

Overall, GallScopeNet is meticulously designed to accurately identify subtle changes in the gallbladder wall and bile ducts, leveraging both normal convolution for detailed feature extraction and coordinate attention for enhanced feature expression. These improvements collectively enable the model to perform robustly and efficiently in the clinical diagnosis of biliary atresia, demonstrating a reduction in false negatives by approximately 2.1% and an increase in true positives by 2.3%, further underscoring its reliability in clinical settings.

### 3.4 Model evaluation criteria

This study constructed a comprehensive evaluation system to rigorously measure the performance of the deep learning model in analyzing ultrasound images for gallbladder abnormalities. This multi-dimensional evaluation system integrates several key metrics, including accuracy (ACC), precision, recall, F1 score, and Matthews correlation coefficient (MCC), as well as core evaluation tools like area under the curve (AUC) and confusion matrix (42–44). This approach is consistent with methodologies employed in recent studies on machine learning models for medical condition identification, ensuring comprehensive evaluation and comparison (45). The goal is to comprehensively test the model’s predictive accuracy, classification comprehensiveness, and consistency with actual diagnoses, ensuring thorough and broad evaluation.

Accuracy, as a fundamental classification performance metric, reveals the correctness of model classification. The calculation of accuracy is shown in Equation 1:

(1) $$\mathrm{Accuracy}=\frac{TP+TN}{TP+TN+FP+FN}$$

where TP (true positive) is true positive, TN (true negative) is true negative, FP (false positive) is false positive, and FN (false negative) is false negative. High accuracy indicates overall good classification tendency.

Precision measures the proportion of true positives among the predicted positive samples, indicating the prediction accuracy for positive cases. The calculation of precision is illustrated in Equation 2:

(2) $$\mathrm{Precision}=\frac{TP}{TP+FP}$$

High recall ensures that all positive cases are captured, thereby reducing missed diagnoses.

Recall focuses on the proportion of actual positives correctly identified, measuring the model’s capability to capture positive cases. The calculation of recall is given in Equation 3:

(3) $$\mathrm{Recall}=\frac{TP}{TP+FN}$$

High recall ensures that all positive cases are captured, thereby reducing missed diagnoses.

The F1 score, as the harmonic mean of precision and recall, balances both metrics and is suitable for imbalanced class situations. The calculation of the F1 score is presented in Equation 4:

(4) $$F1\;\mathrm{score}=2\times \frac{\mathrm{Precision}\times \mathrm{Recall}}{\mathrm{Precision}+\mathrm{Recall}}$$

A high F1 score indicates a balanced precision and recall.

The Matthews correlation coefficient (MCC) is a robust indicator of classification quality, particularly for imbalanced distributions. It ranges from −1 to +1, where +1 signifies perfect classification, 0 represents random classification, and −1 indicates incorrect classification. The calculation of MCC is demonstrated in Equation 5:

(5) $$MCC=\frac{TP\times TN-FP\times FN}{\sqrt{\left(TP+FP\right)\left(TP+FN\right)\left(TN+FP\right)\left(TN+FN\right)}}$$

MCC objectively reflects model performance on imbalanced data.

AUC, the area under the receiver operating characteristic curve, measures classifier performance across all thresholds. Ranging from 0 to 1, a value close to 1 indicates strong distinction between positive and negative cases. AUC is important for reflecting the model’s discrimination ability, considering different false alarm costs.

Confusion matrix visually shows the relationship between model predictions and actual labels through TP, TN, FP, and FN, directly calculating metrics and revealing error patterns, identifying overfitting, underfitting, and biases, guiding model adjustment and optimization.

These metrics are particularly appropriate for the task of biliary atresia diagnosis. Given the critical nature of biliary atresia, where early and accurate detection is essential for effective treatment, each metric provides valuable insights into different aspects of model performance. Accuracy offers a broad measure of correct classifications, ensuring that the model performs well overall. Precision is crucial in minimizing false positives, which is important in a clinical setting to avoid unnecessary treatments or procedures. Recall ensures that true cases of biliary atresia are identified, reducing the risk of missed diagnoses, which could lead to severe complications if left untreated. The F1 score balances precision and recall, offering a comprehensive view of the model’s performance, particularly important in the medical field where both metrics are critical. The MCC provides a robust measure of performance even in the presence of class imbalance, reflecting the model’s capability to correctly classify both positive and negative cases in a balanced manner. The AUC evaluates the model’s ability to distinguish between biliary atresia and non-biliary atresia cases across various thresholds, providing a nuanced understanding of the model’s diagnostic power. Finally, the confusion matrix offers a detailed breakdown of prediction outcomes, allowing for the identification of specific types of errors and guiding further improvements to enhance the model’s accuracy and reliability. Together, these metrics ensure a comprehensive, rigorous, and clinically relevant evaluation of the model’s performance in diagnosing biliary atresia, aligning well with the goals and requirements of our study.

## 4 Results

### 4.1 Experiment

This study constructed a rigorous experimental scheme to comprehensively verify the proposed model’s efficiency, accuracy, and generalization capability across different tasks. The experimental design adopted a five-fold cross-validation mechanism and included an additional test set not involved in training for comprehensive model performance evaluation. In the five-fold cross-validation process, the dataset was evenly divided into five subsets, with each round selecting a different subset as the validation set and the remaining four as the training set. The cycle continued for five rounds, and the results from all rounds were aggregated to obtain the mean performance of the model, reducing accidental biases and enhancing the robustness of the evaluation.

To ensure optimal model performance, parameter optimization was performed using two key techniques: grid search and Bayesian optimization. Grid search involves exhaustively searching through a manually specified subset of the hyperparameter space of the model. This method allows for a comprehensive evaluation of different hyperparameter combinations, ensuring that the best parameters are selected based on model performance. On the other hand, Bayesian optimization is a more sophisticated approach that builds a probabilistic model of the function mapping hyperparameters to the objective and uses this model to select the most promising hyperparameters to evaluate in the true objective function. This technique is particularly useful for optimizing hyperparameters in large, complex models where grid search would be computationally prohibitive.

The impact of these optimization techniques on the model’s performance was significant. Grid search provided a thorough evaluation of hyperparameter combinations, ensuring that the model operates under the best possible settings. Bayesian optimization further refined this process by efficiently navigating the hyperparameter space and identifying optimal parameters that might be missed by grid search. These combined techniques resulted in enhanced model accuracy, robustness, and generalization capability.

During model training, the changes in the loss function were closely monitored. If the training loss plateaued and there was no significant improvement in the validation set performance, it was determined that the model had converged, and training was halted. This typically occurred after approximately 100 iterations, indicating that the model had reached an optimal point where further training would not yield significant performance gains.

The model training process was supported by advanced hardware, including the NVIDIA RTX 3090 GPU and the high-performance Intel i9 CPU, providing the computational power necessary for rapid deep learning model training. The software environment utilized Python 3.9 and the latest stable version of the Pytorch framework 1.12.0, ensuring smooth execution and reproducibility of the experiments.

### 4.2 Comparison experiments

#### 4.2.1 Performance comparison and analysis

##### 4.2.1.1 Performance metrics comparison

**Figure 4:**
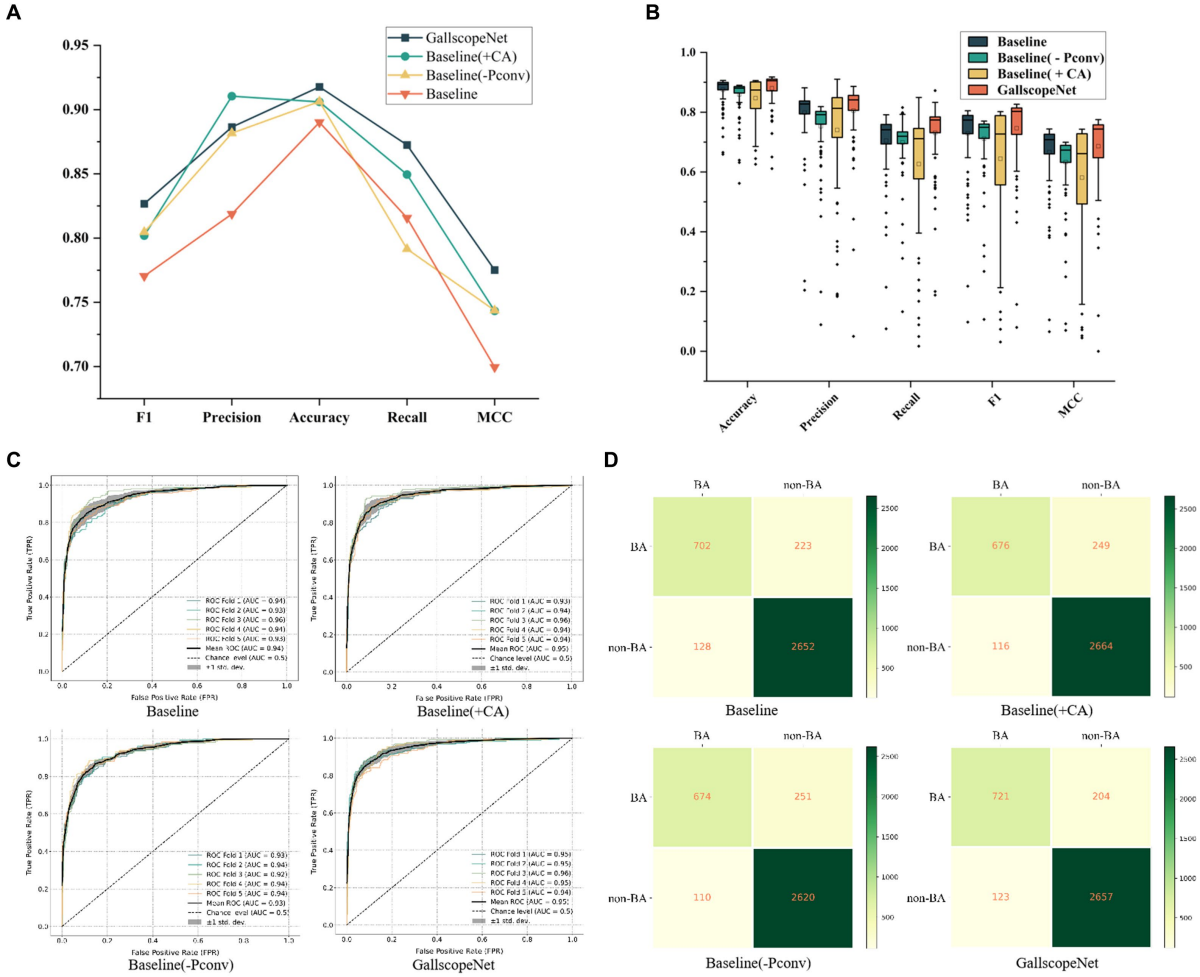
**(A)** Comparison of comprehensive performance indicators for different models, summarizing key metrics. **(B)** Performance and stability comparison of different models across various iteration periods, highlighting consistency and reliability. **(C)** ROC curve comparison of different models following five-fold cross-validation, illustrating the models’ ability to distinguish between positive and negative cases. **(D)** Confusion matrix comparison of different models after five-fold cross-validation, showing classification accuracy through true positives, true negatives, false positives, and false negatives.

In the ablation experiments of GallScopeNet, first, the average indicators after five-fold cross-validation of GallScopeNet compared with other models indicate that GallScopeNet exhibits significant advantages in key indicators such as accuracy, precision, recall, F1 score, and MCC (Figure 4A). This implies that GallScopeNet surpasses comparative models in classification accuracy, precision, recognition ability, balanced comprehensive classification, and classification quality.

##### 4.2.1.2 Stability across epochs

Additionally, the performance and stability comparison of different models across different epochs in the five-fold cross-validation average shows that, although the box plot of GallScopeNet may not have the widest data distribution range, its mean is significantly higher than other models (Figure 4B), indicating that GallScopeNet maintains more stable and high-level performance across multiple epochs.

##### 4.2.1.3 ROC curve analysis

Finally, the ROC curve comparison of various models after five-fold cross-validation indicates that GallScopeNet exhibits the highest AUC value (0.95) within a small curve fluctuation range (Figure 4C), demonstrating GallScopeNet’s strongest ability to distinguish between positive and negative cases and its optimal misdiagnosis capability, maintaining a high true positive rate even with a small error rate. The confusion matrix of GallScopeNet clearly shows the best classification effect, particularly the increase in true positives and true negatives (Figure 4D), indicating that GallScopeNet can more accurately identify BA and non-BA cases in practical applications, reducing misdiagnosis and missed diagnosis.

Overall, GallScopeNet performs exceptionally well in all key performance indicators of the five-fold cross-validation, emphasizing its potential in ultrasound diagnosis of BA.

#### 4.2.2 Model complexity analysis

While GallScopeNet achieved breakthroughs in performance, from the perspective of model complexity, it did not significantly increase in Params (parameter count), FLOPs (floating-point operations), and processing speed (FPS) (Table 1). Despite abandoning the PConv design and introducing innovative coordinate attention, GallScopeNet did not significantly increase the model complexity burden, maintaining efficiency in these key indicators, demonstrating the lightweight design and computational optimization.

**Table 1:** Comparison of complexity indicators of different models in ablation experiments.

Model	Params (M)	FLOPs (G)	FPS
Baseline	21.28	7.33	0.1214
Baseline (+CA)	21.45	7.35	0.1506
Baseline (−PConv)	21.30	7.34	0.1386
GallscopeNet	21.42	7.35	0.1395

#### 4.2.3 Iterative performance analysis

In the iterative performance analysis of the model, GallScopeNet demonstrated its stability and convergence efficiency during the training process, further highlighting its excellent performance.

##### 4.2.3.1 Accuracy stability

**Figure 5:**
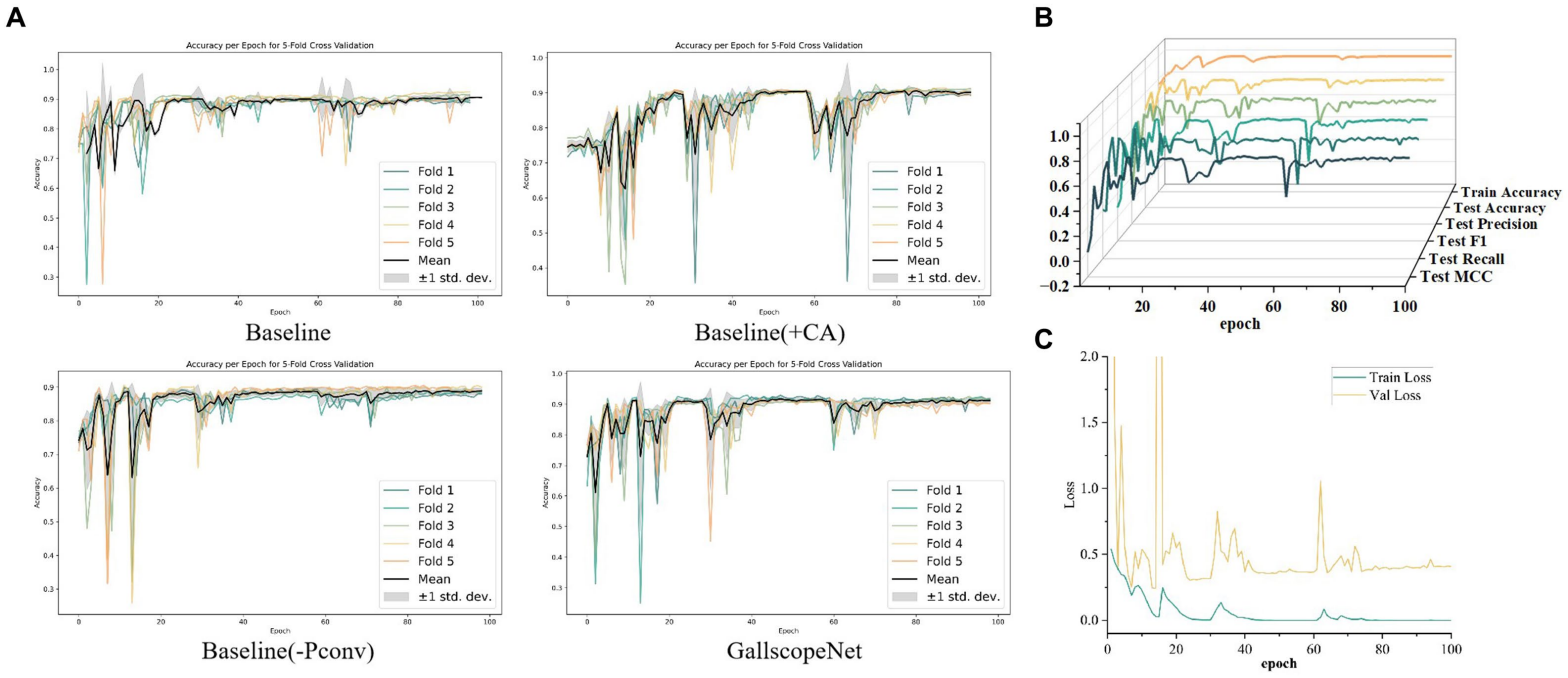
**(A)** Iteration graph of accuracy for different models, showcasing the accuracy trends across iterations. **(B)** Iteration graph of various training and validation metrics for GallScopeNet, demonstrating the convergence and performance consistency. **(C)** Iteration graph of loss values for GallScopeNet during training and validation, highlighting the model’s ability to reduce prediction error over time.

Firstly, in the iteration graph of five-fold cross-validation accuracy, GallScopeNet shows very small variance in the late iteration stages of each fold, indicating highly consistent performance across different data subsets. GallScopeNet quickly reaches stable high accuracy, with minimal differences between folds (Figure 5A). This demonstrates GallScopeNet’s good generalization performance on different data splits, showing stable accuracy during iterations and reducing inter-dataset fluctuations.

##### 4.2.3.2 Convergence of key metrics

Secondly, in the detailed iteration graphs of training and validation metrics, key indicators such as accuracy, precision, recall, F1 score, and MCC for GallScopeNet all show clear convergence trends (Figure 5B). This indicates that the model not only improves performance during training but also consistently optimizes evaluation performance on the validation set, proving GallScopeNet’s effectiveness and generalization ability during training.

##### 4.2.3.3 Loss function analysis

Lastly, in the iteration graph of training and validation loss, GallScopeNet clearly shows a decreasing and stabilizing trend in loss values (Figure 5C). This indicates that the model gradually reduces prediction errors during training, optimizing the gap between model predictions and actual values, achieving a good fitting status. This further emphasizes that GallScopeNet not only quickly finds the optimal solution during training but also maintains good generalization during validation, reflecting the model’s efficient learning capability and stable convergence.

In summary, GallScopeNet’s performance during iterative training, whether in terms of accuracy stability in five-fold cross-validation or the convergence of key metrics and loss functions, fully demonstrates its superiority in learning efficiency and generalization performance. The model quickly converges at different iteration stages and maintains consistent performance on the validation set, highlighting GallScopeNet’s robust, efficient, and accurate potential in ultrasound gallbladder image diagnosis tasks.

### 4.3 External independent testing

To validate the generalization performance of GallScopeNet, 842 images were initially separated from the original dataset to serve as an independent external dataset. This external testing phase was crucial for assessing how well the model performs on unseen data, ensuring its reliability in real-world diagnostic applications.

**Figure 6:**
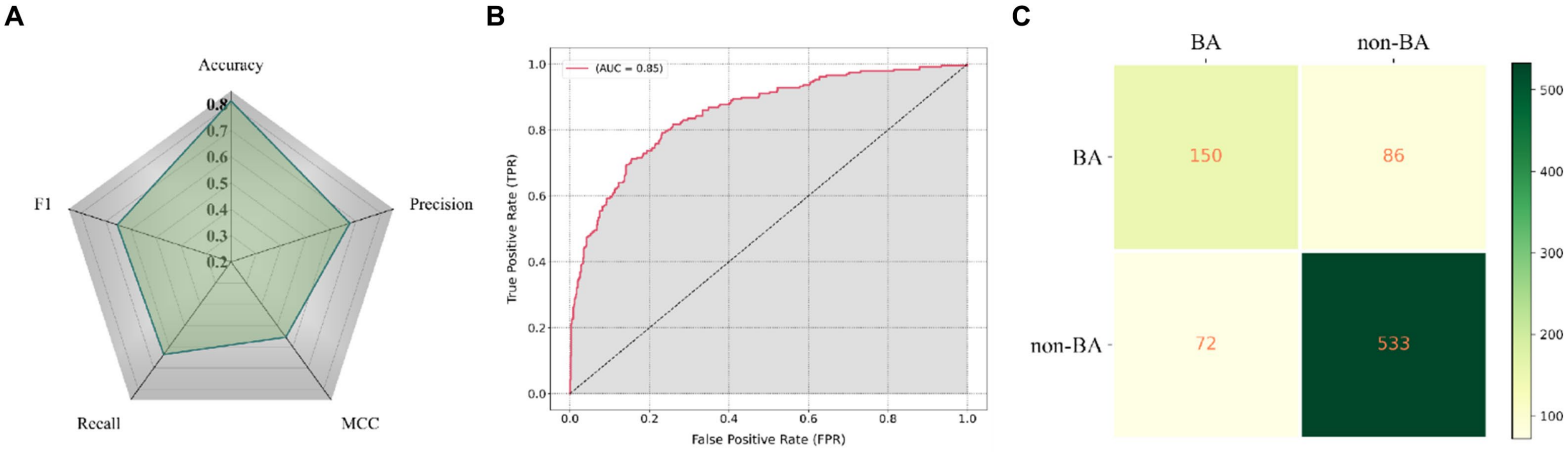
**(A)** Radar chart displaying the comprehensive performance indicators of GallScopeNet on the external independent dataset, effectively summarizing multiple key metrics in a single visualization. **(B)** ROC curve illustrating GallScopeNet’s performance in distinguishing between positive and negative cases on the external independent dataset. **(C)** Confusion matrix visualization depicting the classification performance of GallScopeNet on the external independent dataset.

Firstly, GallScopeNet’s radar chart of indicators demonstrates its comprehensive performance on the independent dataset (Figure 6A). GallScopeNet maintained high levels in key performance indicators such as accuracy, precision, recall, F1 score, and MCC on the new dataset, reflecting the model’s generalization capability. This indicates that GallScopeNet not only performs excellently in training and validation but also maintains high accuracy and stability on unseen data, proving its potential for reliable diagnostic applications. Particularly, the accuracy of 81.21% highlights GallScopeNet’s effectiveness in correctly classifying cases.

Secondly, GallScopeNet achieved an AUC of 0.85 on the external independent dataset (Figure 6B). This value indicates strong performance in distinguishing between positive and negative cases, even on external data, demonstrating high diagnostic accuracy and low misdiagnosis capability. The high AUC value further confirms GallScopeNet’s discrimination ability on external data, proving its generalization capability.

Finally, the confusion matrix results of GallScopeNet on the external data show excellent performance (Figure 6C). The confusion matrix shows high true positives (TP) and true negatives (TN) while maintaining low false positives (FP) and false negatives (FN), indicating that GallScopeNet accurately classifies gallbladder and non-BA cases on new data, reducing misdiagnosis and missed diagnosis. This proves GallScopeNet’s accurate classification ability on external data, reducing the risk of misdiagnosis and missed diagnosis.

Overall, the balanced maintenance of comprehensive indicators, high AUC value, and excellent confusion matrix results demonstrate that GallScopeNet not only performs excellently in training and validation datasets but also has high efficiency and accuracy in diagnosing BA on external datasets, reflecting the model’s reliability and practical value.

### 4.4 Comparison of complexity and accuracy between GallScopeNet and other mainstream models

In a comprehensive comparative evaluation against mainstream models, GallScopeNet demonstrates its superiority in both accuracy and efficiency (Table 2). The comparison focused on several key parameters: accuracy (ACC), the number of integrated parameters (Params, M), floating-point operations per second (FLOPs, G), and frames per second (FPS). These metrics provide a clear picture of both the performance and complexity of the models evaluated.

**Table 2:** Comparison of complexity and accuracy between GallScopeNet and other mainstream models.

Model	Acc (%)	Params (M)	FLOPs (G)	FPS
Vgg16	81.03	134.28	31.0	0.1901
ResNet50	80.98	23.51	8.22	0.1403
ResNet34	80.60	21.29	7.34	0.1349
ResNet18	76.69	11.18	3.64	0.1182
DenseNet	79.81	13.95	5.74	0.1751
GallscopeNet	81.21	21.42	7.35	0.1395

GallScopeNet achieves an accuracy of 81.21%, which is higher than other mainstream models such as ResNet50 (80.98%), ResNet34 (80.60%), DenseNet (79.81%), and Vgg16 (81.03%). The ability of GallScopeNet to capture intricate details and subtle pathological features in ultrasound images contributes to its superior diagnostic performance.

Moreover, GallScopeNet possesses a clear efficiency advantage, reflected in its parameter count of 21.42M, which is comparable to ResNet34’s 21.29M and significantly lower than Vgg16’s 134.28M. Additionally, GallScopeNet requires 7.35G FLOPs, which is less than ResNet50’s 8.22G and comparable to ResNet34’s 7.34G. This lower computational demand underscores its efficiency, making GallScopeNet suitable for deployment in environments with limited processing power. The model also demonstrates impressive speed, processing at 0.1395 frames per second (FPS), which is competitive with ResNet50 (0.1403 FPS) and ResNet34 (0.1349 FPS), while significantly outperforming Vgg16 (0.1901 FPS). This high FPS rate is particularly beneficial for real-time diagnostic applications, where quick and accurate analysis is crucial.

In summary, GallScopeNet not only surpasses other models in terms of accuracy but also optimizes resource utilization through its efficient architectural design. The model’s competitive parameter count, lower FLOPs, and high FPS collectively ensure that it delivers high diagnostic accuracy while being computationally efficient. This makes GallScopeNet particularly advantageous in real-time processing scenarios and resource-limited settings, establishing it as a new benchmark in the field of ultrasound image analysis for biliary atresia diagnosis.

### 4.5 Visualization

**Figure 7:**
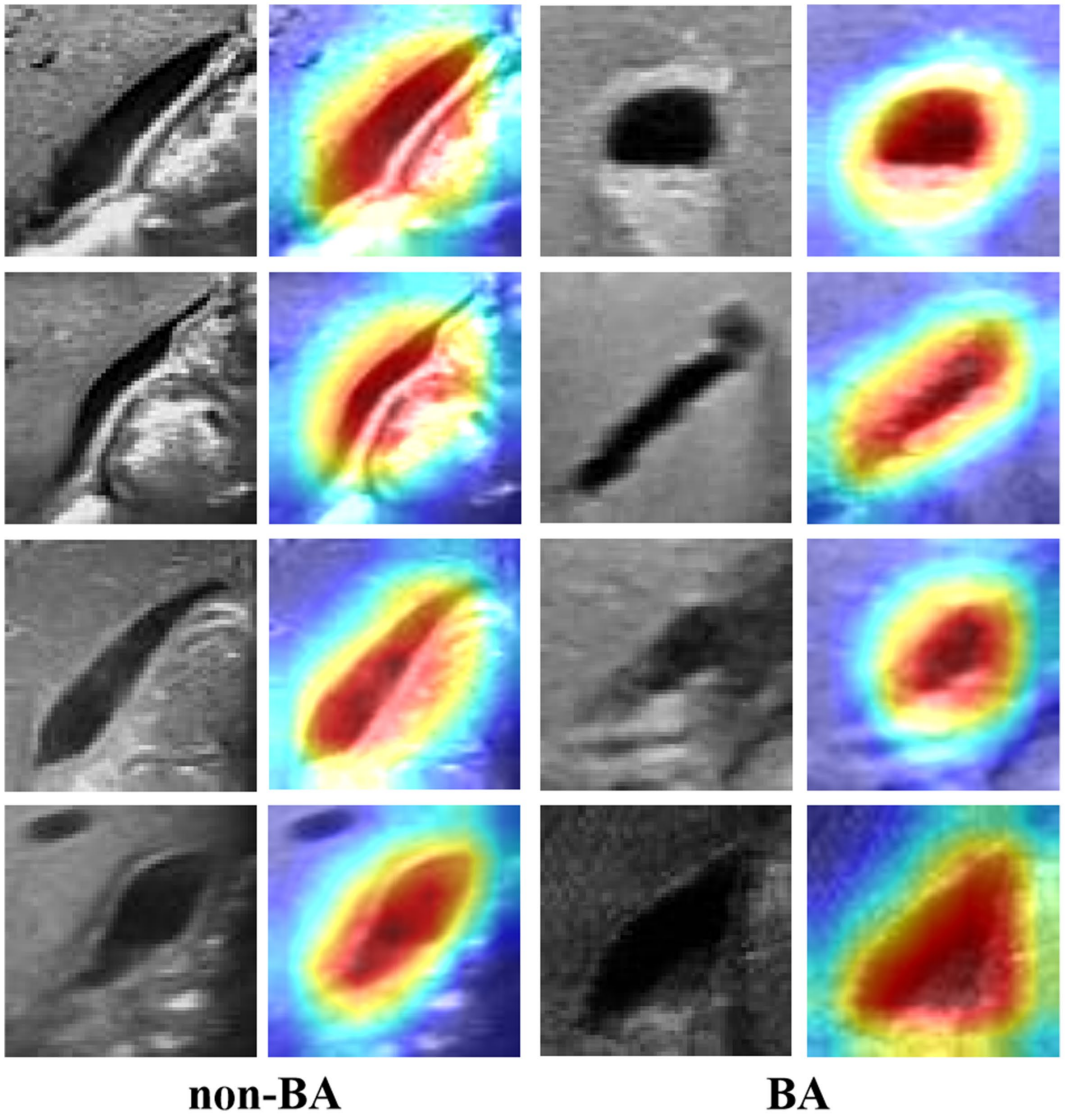
Accurate characterization visualization of GallScopeNet for gallbladder wall features in BA and non-BA states.

To comprehensively evaluate the deep learning model GallScopeNet’s performance in analyzing ultrasound images of BA and non-BA, a detailed feature visualization analysis was conducted. This process involved importing a carefully selected series of BA and non-BA ultrasound images into the GallScopeNet model, aiming to visually demonstrate the model’s ability to capture key pathological markers through the generated feature interest heatmaps (Figure 7). The results showed that GallScopeNet demonstrated high accuracy, accurately marking key pathological features such as gallbladder wall thickening and abnormal blood flow in BA images while successfully recognizing and highlighting normal anatomical structures and physiological blood flow patterns in non-BA images. The significant differences in heatmaps between the two clearly demonstrated the model’s differential diagnostic capabilities. This feature visualization practice not only confirmed GallScopeNet’s ability to effectively extract key information in ultrasound image analysis but also further revealed its decision logic in distinguishing complex pathology from normal states, providing strong empirical support for the model’s efficiency and reliability in the auxiliary diagnosis of BA.

## 5 Discussion

### 5.1 Research summary

This study conducted an in-depth exploration of diagnosing BA from ultrasound images using the deep learning model GallScopeNet. The core of the research lies in enhancing early diagnostic accuracy and model design to reduce dependence on experience. By integrating multi-scale feature extraction strategies and innovative coordinate attention mechanisms, this study effectively addressed the limitations of traditional methods in recognizing subtle pathological features. The introduction of GallScopeNet not only optimized model design but also underwent rigorous validation, ensuring robust performance.

### 5.2 Novelty and significance

This study’s application of deep learning technology to BA diagnosis showcases uniqueness and innovation in two main aspects: firstly, the optimized design of the baseline model by replacing partial convolution with normal convolution. This adjustment, despite increasing computational complexity, significantly enhanced performance, overcoming the model’s limitations in capturing detailed features in complex ultrasound images, particularly in recognizing gallbladder wall and hemodynamic changes (34, 46). Secondly, the innovation lies in introducing coordinate attention (CA), embedding positional information into feature extraction. This mechanism, building upon traditional channel attention, allows the model to consider spatial dependencies and precise positional details along both horizontal and vertical directions. GallScopeNet, integrated with CA, not only exhibits precision in complex image processing but also advances the application boundaries of deep learning in BA diagnosis, introducing new possibilities to the field.

### 5.3 Clinical significance

The development of GallScopeNet holds significant clinical implications for the early diagnosis of BA. By reducing dependence on operators, the model enhances diagnostic accuracy and consistency, shortening identification time. For patients, this means earlier initiation of treatment plans, reducing complication risks, improving prognosis, and increasing survival rates (47). Compared to traditional ultrasound analysis by radiologists, GallScopeNet offers a more standardized and objective approach, minimizing the variability that arises from human interpretation. Studies have shown that traditional imaging diagnostic methods for biliary atresia, such as hepatobiliary scintigraphy, typically achieve a specificity ranging between 70 and 80% (48, 49). In contrast, the specificity of the GallScopeNet model used in this study is 86.10%, indicating a superior diagnostic performance and demonstrating its potential to reduce misdiagnosis in clinical practice. This makes GallScopeNet particularly valuable in settings where experienced radiologists are not available, ensuring a high level of diagnostic reliability across different healthcare providers. In resource-limited settings, GallScopeNet’s efficiency is especially critical, providing a feasible diagnostic tool for remote or resource-constrained medical institutions (10).

The advanced technology employed in GallScopeNet, when combined with the insights and oversight of experienced medical professionals, can achieve exceptional efficiency and accuracy in the diagnosis of biliary atresia. While the model offers significant advantages in terms of diagnostic consistency and objectivity, the role of medical doctors remains crucial. Their supervision ensures that the deep learning model’s outputs are appropriately interpreted within the broader context of each patient’s unique clinical scenario. This collaborative approach enhances the reliability of diagnoses, ensuring that technological advancements complement, rather than replace, the critical expertise of healthcare providers.

### 5.4 Clinical integration

Integrating GallScopeNet into the clinical workflow is essential for its effective application in diagnosing biliary atresia (BA) and other biliary diseases. This involves seamless integration with existing hospital information systems (HIS) and picture archiving and communication systems (PACS) for real-time access to ultrasound images and patient data. Developing a user-friendly interface will allow clinicians to easily interact with GallScopeNet, upload images, view results, and receive diagnostic suggestions without extensive technical expertise. Providing clinical training and ongoing support will help clinicians understand and trust the model’s results, effectively incorporating them into their diagnostic processes. Obtaining regulatory approval through rigorous testing will establish the model’s safety and efficacy. Continuous feedback from clinicians will be vital for iterative improvements. Regular monitoring and evaluation will track diagnostic outcomes and identify areas for enhancement, ensuring high standards of accuracy and reliability.

However, there are potential challenges and limitations in real-world clinical settings. Ensuring consistent quality and standardization of ultrasound images across different facilities is a major challenge, as variations in equipment and protocols can affect performance. Integration with existing HIS and PACS systems may face technical hurdles, requiring IT infrastructure adjustments. Clinicians may need time to adapt to and trust AI-based tools, necessitating robust training programs. The model’s reliance on high-quality input data means suboptimal images could lead to inaccurate predictions, requiring effective quality control. Navigating the regulatory landscape for approval can be complex and time-consuming, potentially delaying deployment. Addressing these challenges is crucial for the successful implementation and adoption of GallScopeNet in clinical practice.

### 5.5 Limitations

#### 5.5.1 Data diversity and limitations

The current dataset, although covering confirmed and non-BA cases, lacks a wide age range, racial diversity, geographical spread, disease duration, and severity levels, limiting the model’s generalizability and applicability. Future work should integrate more diverse data to encompass different population characteristics and improve the model’s comprehensiveness.

#### 5.5.2 Model complexity and efficiency balance

Although the introduction of normal convolution and coordinate attention improved feature extraction capabilities, the increased parameter count and computational complexity present challenges for resource-constrained edge devices or real-time diagnostic scenarios. Future work should continue to optimize the model architecture, such as using lightweight techniques, to maintain performance while reducing resource consumption.

#### 5.5.3 Lack of long-term follow-up data

The study lacks long-term follow-up data, making it difficult to evaluate the model’s impact on patient prognosis and quality of life. Future work should collect long-term follow-up data to assess the model’s long-term effects on patient prognosis and verify its practical value in clinical decision-making.

#### 5.5.4 Sensitivity to specific pathological features

Although the model has high recognition capabilities for BA, its differentiation of other biliary diseases such as inflammation, infection, stones, and cysts needs further optimization. Future work should focus on improving the model’s accuracy in identifying different pathological conditions to enhance diagnostic comprehensiveness.

### 5.6 Future directions

In advancing GallScopeNet’s research on ultrasound diagnosis of BA, this study focuses on several practical directions: primarily refining data processing techniques by incorporating advanced image enhancement and dynamic compensation technologies to optimize feature extraction strategies, adapting to different ultrasound images, and improving image resolution and accuracy (50). Secondly, expanding the model’s practical applications by integrating domain adaptation learning and diverse tasks, such as diagnosing other biliary diseases beyond BA, enhancing the model’s adaptability and applicability across various cases (51). Additionally, conducting large-scale clinical validation studies to ensure the model’s diagnostic accuracy and clinical relevance in real-world settings, facilitating the transition of laboratory findings to clinical practice and practical applications (52–54).

Future research should also explore the integration of GallScopeNet with other diagnostic modalities, such as combining ultrasound with MRI or CT imaging, to provide a more comprehensive diagnostic tool. This multimodal approach could further improve diagnostic accuracy and provide a more holistic view of the patient’s condition. Moreover, leveraging federated learning techniques could enable the model to be trained on data from multiple institutions without compromising patient privacy, thereby increasing the robustness and generalizability of GallScopeNet across diverse populations and settings.

In summary, GallScopeNet’s future emphasizes not only technical refinement but also practical application, combined with clinical practice, to advance the practical implementation of ultrasound image diagnosis for BA (54). By addressing these areas, GallScopeNet can evolve into a more versatile and powerful tool, ultimately improving patient outcomes and advancing the field of medical diagnostics.

## 6 Conclusion

This study explored the diagnosis of BA, a severe neonatal health threat, using deep learning technology, particularly the innovative design and application of the GallScopeNet model, achieving significant progress in ultrasound image diagnosis. By analyzing a carefully constructed dataset of gallbladder ultrasound images, this study not only optimized the feature extraction process, enhancing the model’s learning performance in complex ultrasound images, but also achieved notable improvements in diagnostic accuracy and generalization capabilities. GallScopeNet’s design highlights two key improvements: firstly, replacing partial convolution with normal convolution, which, despite sacrificing some computational efficiency, significantly improved the model’s sensitivity to subtle structural features, especially in capturing gallbladder wall and hemodynamic changes; secondly, integrating coordinate attention, maintaining model efficiency while enhancing feature expression and optimizing the recognition of gallbladder and bile duct structures. These improvements, while keeping model complexity manageable, significantly enhance the identification of subtle changes in the gallbladder wall and bile ducts, crucial for BA diagnosis.

Through comprehensive evaluation using five-fold cross-validation and independent testing, GallScopeNet performed exceptionally well across key indicators such as accuracy, precision, recall, F1 score, confusion matrix, and AUC. On external test datasets, the model maintained high classification performance, achieving an accuracy of 81.21% and an AUC of 0.85, reducing misdiagnosis and missed diagnosis, proving its reliability and practical value. Visualization analysis clearly demonstrated the model’s ability to capture key pathological features in BA and non-BA images, further validating its diagnostic capabilities.

This study is one of the first attempts to use a lightweight, efficient deep learning model tailored for embedded systems in the context of BA diagnosis. GallScopeNet achieves a high level of accuracy while being optimized for computational efficiency, making it suitable for use in embedded systems and resource-constrained environments. This balance between model complexity and performance ensures that the tool can be widely deployed, offering significant benefits in early diagnosis and treatment planning for BA.

The development and application of GallScopeNet represent a breakthrough in ultrasound image diagnosis of BA, particularly in recognizing subtle features and analyzing complex structures. Through innovative model design and rigorous validation, GallScopeNet not only demonstrates efficiency and accuracy in BA diagnosis but also maintains generalizability, providing a reliable tool for clinical applications, especially in resource-limited settings.

Future efforts to optimize and expand data diversity, improve model efficiency, collect long-term follow-up data, and extend the model to more biliary diseases will deepen GallScopeNet’s potential, advancing the precision medical practice of ultrasound diagnosis for BA and beyond. By addressing these areas, GallScopeNet can evolve into a more versatile and powerful tool, ultimately improving patient outcomes and advancing the field of medical diagnostics.

## Data Availability

The original contributions presented in the study are included in the article/supplementary material, further inquiries can be directed to the corresponding author.

## References

[1] Hartley JL Davenport M Kelly DA. Biliary atresia. Lancet. (2009) 374:1704–13. doi: 10.1016/S0140-6736(09)60946-6 19914515

[2] Zeng X Liao Y Qiao X Liang K Luo Q Deng M. Novel NIR-II fluorescent probes for biliary atresia imaging. Acta Pharm Sin B. (2023) 13:4578–90. doi: 10.1016/j.apsb.2023.07.005, PMID: 37969732 PMC10638547

[3] Zhou L Shan Q Tian W Wang Z Liang J Xie X. Ultrasound for the diagnosis of biliary atresia: a meta-analysis. Am J Roentgenol. (2016) 206:W73–82. doi: 10.2214/AJR.15.15336, PMID: 27010179

[4] Banc-Husu AM Bass LM. Transient elastography in pediatric liver disease. J Pediatr Gastroenterol Nutr. (2021) 73:141–4. doi: 10.1097/MPG.0000000000003168 34016882

[5] Barr RG Ferraioli G Palmeri ML Goodman ZD Garcia-Tsao G Rubin J. Elastography assessment of liver fibrosis: Society of Radiologists in Ultrasound Consensus Conference Statement. Ultrasound Q. (2016) 32:94–107. doi: 10.1097/RUQ.0000000000000209 27233069

[6] Lien TH Chang MH Wu JF Chen HL Lee HC Chen AC. Effects of the infant stool color card screening program on 5-year outcome of biliary atresia in Taiwan. Hepatology. (2011) 53:202–8. doi: 10.1002/hep.24023, PMID: 21140377

[7] Harpavat S Garcia-Prats JA Anaya C Brandt ML Lupo PJ Finegold MJ. Diagnostic yield of newborn screening for biliary atresia using direct or conjugated bilirubin measurements. JAMA. (2020) 323:1141–50. doi: 10.1001/jama.2020.0837, PMID: 32207797 PMC7093763

[8] Yoon H Lim HJ Kim J Lee M. Diagnostic imaging of biliary atresia. J Korean Soc Radiol. (2022) 83:991–1002. doi: 10.3348/jksr.2022.0077, PMID: 36276203 PMC9574267

[9] Sandberg JK Sun Y Ju Z Liu S Jiang J Koci M. Ultrasound shear wave elastography: does it add value to gray-scale ultrasound imaging in differentiating biliary atresia from other causes of neonatal jaundice? Pediatr Radiol. (2021) 51:1654–66. doi: 10.1007/s00247-021-05024-9, PMID: 33772640

[10] Zhou W Ye Z Huang G Zhang X Xu M Liu B. Interpretable artificial intelligence-based app assists inexperienced radiologists in diagnosing biliary atresia from sonographic gallbladder images. BMC Med. (2024) 22:29. doi: 10.1186/s12916-024-03247-9, PMID: 38267950 PMC10809457

[11] Masud M Eldin RA Hossain MS. Convolutional neural network-based models for diagnosis of breast cancer. Neural Comput Appl. (2022) 34:11383–94. doi: 10.1007/s00521-020-05394-5, PMID: 33052172 PMC7545025

[12] Esteva A Kuprel B Novoa RA Ko J Swetter SM Blau HM. Dermatologist-level classification of skin cancer with deep neural networks. Nature. (2017) 542:115–8. doi: 10.1038/nature21056, PMID: 28117445 PMC8382232

[13] Khosravi P Kazemi E Imielinski M Elemento O Hajirasouliha I. Deep convolutional neural networks enable discrimination of heterogeneous digital pathology images. EBioMedicine. (2018) 27:317–28. doi: 10.1016/j.ebiom.2017.12.026, PMID: 29292031 PMC5828543

[14] Vivanti R Joskowicz L Lev-Cohain N Ephrat A Sosna J. Patient-specific and global convolutional neural networks for robust automatic liver tumor delineation in follow-up CT studies. Med Biol Eng Comput. (2018) 56:1699–713. doi: 10.1007/s11517-018-1803-6, PMID: 29524116

[15] Zhou W Yang Y Yu C Liu J Duan X Weng Z. Ensembled deep learning model outperforms human experts in diagnosing biliary atresia from sonographic gallbladder images. Nat Commun. (2021) 12:1259. doi: 10.1038/s41467-021-21466-z, PMID: 33627641 PMC7904842

[16] Hsu FR Dai ST Chou CM Huang SY. The application of artificial intelligence to support biliary atresia screening by ultrasound images: a study based on deep learning models. PLoS One. (2022) 17:e0276278. doi: 10.1371/journal.pone.0276278, PMID: 36260613 PMC9581370

[17] Zhou W Zhou L. Ultrasound for the diagnosis of biliary atresia: from conventional ultrasound to artificial intelligence. Diagnostics. (2021) 12:51. doi: 10.3390/diagnostics12010051, PMID: 35054217 PMC8775261

[18] Park W-H Choi SO Lee SK Kim SP Zeon SK Lee SL. A new diagnostic approach to biliary atresia with emphasis on the ultrasonographic triangular cord sign: comparison of ultrasonography, hepatobiliary scintigraphy, and liver needle biopsy in the evaluation of infantile cholestasis. J Pediatr Surg. (1997) 32:1555–9. doi: 10.1016/S0022-3468(97)90451-6, PMID: 9396524

[19] Duan X Yang L Zhu W Yuan H Xu X Wen H. Is the diagnostic model based on convolutional neural network superior to pediatric radiologists in the ultrasonic diagnosis of biliary atresia? Front Med. (2024) 10:1308338. doi: 10.3389/fmed.2023.1308338 PMC10800889 38259860

[20] Gulshan V Peng L Coram M Stumpe MC Wu D Narayanaswamy A. Development and validation of a deep learning algorithm for detection of diabetic retinopathy in retinal fundus photographs. JAMA. (2016) 316:2402–10. doi: 10.1001/jama.2016.17216, PMID: 27898976

[21] Liu Y Jain A Eng C Way DH Lee K Bui P. A deep learning system for differential diagnosis of skin diseases. Nat Med. (2019) 25:1301–9. doi: 10.1038/s41591-019-0508-1, PMID: 32424212

[22] Rajpurkar P Irvin J Ball RL Zhu K Yang B Mehta H. Deep learning for chest radiograph diagnosis: a retrospective comparison of the Che XNeXt algorithm to practicing radiologists. PLoS Med. (2018) 15:e1002686. doi: 10.1371/journal.pmed.1002686, PMID: 30457988 PMC6245676

[23] Zhou W Ye Z Huang G Zhang X Xu M Liu B. Interpretable artificial intelligence-based app assists inexperienced radiologists in diagnosing biliary atresia from sonographic gallbladder images. BMC Med. (2021) 22:29. doi: 10.1186/s12916-021-02011-y PMC10809457 38267950

[24] Gu YH Yokoyama K Mizuta K Tsuchioka T Kudo T Sasaki H. Stool color card screening for early detection of biliary atresia and long-term native liver survival: a 19-year cohort study in Japan. J Pediatr. (2015) 166:897–902.e1. doi: 10.1016/j.jpeds.2014.12.063, PMID: 25681196

[25] Caponcelli E Knisely AS Davenport M. Cystic biliary atresia: an etiologic and prognostic subgroup. J Pediatr Surg. (2008) 43:1619–24. doi: 10.1016/j.jpedsurg.2007.12.058, PMID: 18778995

[26] Zhang Y Gorriz JM Dong Z. Deep learning in medical image analysis. J Imaging. (2021) 7:74. doi: 10.3390/jimaging7040074, PMID: 34460524 PMC8321330

[27] Zenodo. (2021). youngyzzZ/sonographic-gallbladder-images-for-BA-diagnosis: fifth release of my project (v1.0.4). Available at: 10.5281/zenodo.4445734

[28] Zimmerman JB Pizer SM Staab EV Perry JR McCartney W Brenton BC. An evaluation of the effectiveness of adaptive histogram equalization for contrast enhancement. IEEE Trans Med Imaging. (1988) 7:304–12. doi: 10.1109/42.14513, PMID: 18230483

[29] Arias-Castro EDDL Donoho DL. Does median filtering truly preserve edges better than linear filtering? Ann Stat. (2009) 37:1172–206. doi: 10.1214/08-AOS604

[30] Diwakar M Pandey NK Singh R Sisodia D Arya C Singh P. Low-dose COVID-19 CT image denoising using CNN and its method noise thresholding. Curr Med Imaging. (2023) 19:182–93. doi: 10.2174/1573405618666220404162241, PMID: 35379137

[31] Farid H. Blind inverse gamma correction. IEEE Trans Image Process. (2001) 10:1428–33. doi: 10.1109/83.951529, PMID: 18255487

[32] Sibbald M Zwaan L Yilmaz Y Lal S. Incorporating artificial intelligence in medical diagnosis: a case for an invisible and (un) disruptive approach. J Eval Clin Pract. (2024) 30:3–8. doi: 10.1111/jep.13730, PMID: 35761764

[33] Chen J Kao S He H Zhuo W Wen S Lee C. (2023). Run don’t walk: chasing higher FLOPS for faster neural networks. 2023 IEEE/CVF Conference on Computer Vision and Pattern Recognition (CVPR). 17–24 June 2023: Vancouver, BC, Canada.

[34] Yu J Hu Z Sun G Xing X Li F. (2024). A novel channel attention module for integrity and importance of feature map. Fifteenth International Conference on Graphics and Image Processing (ICGIP 2023).

[35] Liu G Dundar A Shih KJ Wang T Reda FA Sapra K. Partial convolution for padding inpainting and image synthesis. IEEE Trans Pattern Anal Mach Intell. (2022) 45:1–15. doi: 10.1109/TPAMI.2022.3209702 36155473

[36] Yan S Zhang X. PCNet: partial convolution attention mechanism for image inpainting. Int J Comput Appl. (2022) 44:738–45. doi: 10.1080/1206212X.2021.1909280

[37] Lin DC Wu KY Sun FJ Huang CC Wu TH Shih SL. A quantitative image analysis using MRI for diagnosis of biliary atresia. Clin Imaging. (2019) 53:186–90. doi: 10.1016/j.clinimag.2018.10.001, PMID: 30415184

[38] Hu M Lin H Fan Z Gao W Yang L Liu C. Learning to recognize chest-Xray images faster and more efficiently based on multi-kernel depthwise convolution. IEEE Access. (2020) 8:37265–74. doi: 10.1109/ACCESS.2020.2974242

[39] Xiao M Yang B Wang S Zhang Z He Y. Fine coordinate attention for surface defect detection. Eng Appl Artif Intell. (2023) 123:106368. doi: 10.1016/j.engappai.2023.106368

[40] Yang W Wu J Zhang J Gao K Du R Wu Z. Deformable convolution and coordinate attention for fast cattle detection. Comput Electron Agric. (2023) 211:108006. doi: 10.1016/j.compag.2023.108006

[41] Yi S Li J Liu X Yuan X. CCAFFMNet: dual-spectral semantic segmentation network with channel-coordinate attention feature fusion module. Neurocomputing. (2022) 482:236–51. doi: 10.1016/j.neucom.2021.11.056

[42] Chicco D Jurman G. The advantages of the Matthews correlation coefficient (MCC) over F1 score and accuracy in binary classification evaluation. BMC Genomics. (2020) 21:6. doi: 10.1186/s12864-019-6413-7 31898477 PMC6941312

[43] Deng X Liu Q Deng Y Mahadevan S. An improved method to construct basic probability assignment based on the confusion matrix for classification problem. Inf Sci. (2016) 340-341:250–61. doi: 10.1016/j.ins.2016.01.033

[44] Powers DM. (2012). The problem of area under the curve. 2012 IEEE International Conference on Information Science and Technology. 23–25 March 2012: Wuhan, China

[45] Kumar K Kumar N Kumar A Mohammed MA Al-Waisy AS Jaber MM. Identification of cardiac patients based on the medical conditions using machine learning models. Comput Intell Neurosci. (2022) 2022:5882144. doi: 10.1155/2022/5882144, PMID: 35909858 PMC9329013

[46] Silver JD Ritchie ME Smyth GK. Microarray background correction: maximum likelihood estimation for the normal-exponential convolution. Biostatistics. (2009) 10:352–63. doi: 10.1093/biostatistics/kxn042, PMID: 19068485 PMC2648902

[47] Chen X Zhao D Ji H Chen Y Li Y Zuo Z. Predictive modeling for early detection of biliary atresia in infants with cholestasis: insights from a machine learning study. Comput Biol Med. (2024) 174:108439. doi: 10.1016/j.compbiomed.2024.108439, PMID: 38643596

[48] Esmaili J Izadyar S Karegar I Gholamrezanezhad A. Biliary atresia in infants with prolonged cholestatic jaundice: diagnostic accuracy of hepatobiliary scintigraphy. Abdom Imaging. (2007) 32:243–7. doi: 10.1007/s00261-006-9049-4, PMID: 16967250

[49] Kianifar HR Tehranian S Shojaei P Adinehpoor Z Sadeghi R Kakhki VRD. Accuracy of hepatobiliary scintigraphy for differentiation of neonatal hepatitis from biliary atresia: systematic review and meta-analysis of the literature. Pediatr Radiol. (2013) 43:905–19. doi: 10.1007/s00247-013-2623-3, PMID: 23519699

[50] Zhang L Zhang J Li Z Song Y. A multiple-channel and atrous convolution network for ultrasound image segmentation. Med Phys. (2020) 47:6270–85. doi: 10.1002/mp.14512, PMID: 33007105

[51] Wang Y Yao Q Kwok JT Ni LM. Generalizing from a few examples: a survey on few-shot learning. ACM Comput Surv. (2020) 53:1–34. doi: 10.1145/3386252

[52] Esteva A Robicquet A Ramsundar B Kuleshov V DePristo M Chou K. A guide to deep learning in healthcare. Nat Med. (2019) 25:24–9. doi: 10.1038/s41591-018-0316-z 30617335

[53] Topol EJ. High-performance medicine: the convergence of human and artificial intelligence. Nat Med. (2019) 25:44–56. doi: 10.1038/s41591-018-0300-7, PMID: 30617339

[54] Jiang F Jiang Y Zhi H Dong Y Li H Ma S. Artificial intelligence in healthcare: past, present and future. Stroke Vasc Neurol. (2017) 2:230–43. doi: 10.1136/svn-2017-000101, PMID: 29507784 PMC5829945

