# Corrigendum: Deep learning-driven ultrasound-assisted diagnosis: optimizing GallScopeNet for precise identification of biliary atresia

**DOI:** 10.3389/fmed.2024.1518391

**Published:** 2024-11-12

**Authors:** Yupeng Niu, Jingze Li, Xiyuan Xu, Pu Luo, Pingchuan Liu, Jian Wang, Jiong Mu

**Affiliations:** ^1^College of Information Engineering, Sichuan Agricultural University, Ya'an, China; ^2^Artificial Intelligence Laboratory, Sichuan Agricultural University, Ya'an, China; ^3^College of Veterinary Medicine, Sichuan Agricultural University, Chengdu, China; ^4^Ya'an People's Hospital, Ya'an, Sichuan, China; ^5^Department of Neurology, The Affiliated Hospital, Southwest Medical University, Luzhou, Sichuan, China

**Keywords:** biliary atresia (BA), deep learning, ultrasound diagnosis, feature extraction, clinical application

In the published article, there was an error regarding the affiliations for Pingchuan Liu and Jian Wang. As well as having affiliation 4, they should also have “Department of Neurology, The Affiliated Hospital, Southwest Medical University, Luzhou, Sichuan, China”.

In the published article, there was an error in the Funding statement. The funding statement originally only included “the Health Commission of Sichuan Province, project number 23LCYJ034” and omitted additional funding sources. The correct Funding statement appears below.

